# Nrf2-Mediated Metabolic Reprogramming in Cancer

**DOI:** 10.1155/2018/9304091

**Published:** 2018-01-29

**Authors:** Yan-Yang Wang, Juan Chen, Xiao-Ming Liu, Ren Zhao, Hong Zhe

**Affiliations:** ^1^Department of Radiation Oncology, General Hospital of Ningxia Medical University, Yinchuan, Ningxia 750004, China; ^2^Cancer Institute, Ningxia Medical University, Yinchuan, Ningxia 750004, China; ^3^Department of Pulmonary and Critical Care Medicine, General Hospital of Ningxia Medical University, Yinchuan, Ningxia 750004, China; ^4^Human Stem Cell Institute, General Hospital of Ningxia Medical University, Yinchuan, Ningxia 750004, China

## Abstract

Metabolic reprogramming is one of the hallmarks of cancer. Nrf2 pathway is one of the critical signaling cascades involved in cell defense and survival against oxidative stress. The significance of Nrf2 in cancer metabolism begins to be recognized. In this minireview, we focus on the Nrf2-mediated cancer metabolic reprogramming and intend to highlight the role of Nrf2 in the regulation of malignant transformation, cancer proliferation, and the development of treatment resistance via metabolic adaptations. We hope for the development of noninvasive biomarkers and novel therapeutic approaches for cancer based on Nrf2-directed cancer metabolic reprogramming in the near future.

## 1. Introduction

Metabolic reprogramming is the one of the hallmarks of cancer [1, 2]. In order to meet the biosynthetic demands of increased proliferation, cancer cells modify core metabolism by increasing key metabolic pathways such as glycolysis, pentose phosphate pathway (PPP), and glutaminolysis [3–9]. These metabolic modifications not only support cancer cells to survive but also interact with oncogenic signaling pathways, such as phosphoinositide 3-kinase/protein kinase B- (PI3K/Akt), Myc-, Ras-, p53-, and reactive oxygen species- (ROS-) related pathways. These interactions enhance the invasive and metastatic properties of cancer cells [10, 11]. As one of the critical components of antioxidative molecules, Nrf2 affects multiple aspects of metabolic reprogramming, including inhibition of lipogenesis, facilitation of flux through the PPP, and increased nicotinamide adenine dinucleotide phosphate (NADPH) regeneration and purine biosynthesis through regulating key metabolic enzymes or affecting the crosstalk with several oncogenic pathways [12–18]. For example, increased glycosylation and glutaminolysis in early-stage lung cancer accompanied by Nrf2 activation was observed in recent study [19], demonstrating a critical role of Nrf2 in metabolic reprogramming of cancer.

The interface between redox and metabolism of cancer has been extensively reviewed before [15]. In this minireview, we focus on the Nrf2-mediated cancer metabolic reprogramming and highlight the role of Nrf2 in the regulation of malignant transformation, cancer proliferation, and development of treatment resistance via metabolic adaptations.

## 2. Nrf2 Signaling Pathway

The Nrf2 signaling pathway has a crucial role in maintaining cellular and tissue homeostasis and protecting cells against electrophilic or oxidative stress [20, 21]. As a cap “n” collar (CNC) transcription factor, Nrf2 can interact with small Maf proteins and bind to the promoters of cytoprotective and antioxidative genes to induce their transcription [22, 23]. Under homeostatic conditions, Nrf2 binds to its cytosolic inhibitor Keap1 and facilitates ubiquitination by the Cullin E3 ligase [24]. In response to stress, the cysteine residues in Keap1 change conformation [25, 26], therefore leading to the separation of Keap1 and Nrf2. As a consequence of the separation, Nrf2 translocates into the nucleus and induces the expression of target genes, such as NADPH quinone oxidoreductase (NQO-1), glutathione S-transferases (GSTs), heme oxygenase-1 (HMOX1), and glutamate-cysteine ligase (GCL) subunits [27, 28]. Moreover, protein which contains the motif that is similar to the ETGE of Nrf2, such as p62, can compete with Nrf2 to bind Keap1 and directly activate Nrf2 [29, 30]. In addition to Keap1-dependent activation, there are other alternative pathways that can impact Nrf2 signaling. For example, protein kinase C (PKC) can directly phosphorylate Nrf2 at Ser40 leading to the upregulation of Nrf2 [31, 32]. Emerging evidence indicates that the activation of Nrf2 can profoundly influence the initiation and progression of cancer [16, 33–35].

## 3. Metabolic Reprogramming in Cancer

In order to support extensive proliferation and sustain the invasive phenotypes, cancer cells need to reprogram their metabolic pathways and energy production networks. This phenomenon is named cancer metabolic reprogramming and observed by Warburg et al. firstly in 1920s [8]. As we know, metabolic reprogramming makes a great contribution to the rapid proliferation of cancer at least via supporting the biosynthetic needs [6, 8, 11]. Apart from the well-known Warburg effect of aerobic glycolysis, several other metabolic adaptations, such as enhancement of mitochondrial biogenesis, elevation of lipid metabolism, and upregulation of glutaminolysis, have been described [2]. Glycolysis is one of the remarkable features of cancer metabolic alternations. Compared to healthy and well-differentiated cells, cancer cells prefer using glycolysis even in the presence of oxygen, thereby producing lactate and cutting down the use of the tricarboxylic acid (TCA) cycle [36]. In addition to glycolysis, many tumors also fuel their cellular bioenergetics and metabolism through glutaminolysis. Glutaminolysis catabolizes glutamine as a carbon donor, not only for adenosine triphosphate (ATP) but also for biosynthesis and rapid proliferation of cancer [37]. Oncogenic activation also elevates mitochondrial metabolism to produce ATP and TCA cycle intermediates used as precursors for biosynthesis [38]. Another major change in cancer metabolism is upregulation of lipid metabolism. In fact, increased lipogenesis is considered a critical characteristic of many cancers, with de novo fatty acid (FA) synthesis supporting membrane biogenesis, as well as the energetic demands of rapid proliferation [39]. Nrf2 is a transcription factor that activates the transcription of antioxidant genes as indicated previously. More and more evidences suggest that Nrf2 signaling pathways represent regulatory mediators of the perturbed metabolic activities of various cancer cells [16, 40].

## 4. Nrf2-Mediated Metabolic Reprogramming in Cancer

### 4.1. Malignant Transformation

Caveolin-1 (Cav-1), a scaffold protein, can be modulated by elevated ROS and contributes to the regulation of cellular responses to environmental cues [41]. Hart et al. [42] found that Cav-1, Nrf2, and Keap1 could bind together and form a ternary complex. The suppression of Cav-1 by any cause or oxidative stress itself can promote the disassembly of the Nrf2/Cav-1/Keap1 complex, persistent Nrf2 activation, and the upregulation of manganese superoxide dismutase (MnSOD). Enhancement of MnSOD expression activated adenosine 5′-monophosphate- (AMP-) activated protein kinase (AMPK), which led to glycolytic switch and malignant transformation. On the contrary, rescued Cav-1 expression in a breast cancer cell line suppressed Nrf2 and downregulated MnSOD. Clinical data also confirmed that decreased Cav-1 expression was associated with high tumor grade and low 5-year survival of breast cancer. These results revealed the role of Cav-1 and Nrf2 in metabolic reprogramming and breast malignant transformation.

Kowalik et al. [43] investigated the value of metabolic reprogramming in the transformation of early preneoplastic foci to hepatocellular carcinoma (HCC). The suppression of oxidative phosphorylation (OXPHOS) and enhancement of glucose utilization to fuel the PPP were observed during the malignant transformation. In addition, they also found that Nrf2 triggers the activation of glucose-6-phosphate dehydrogenase (G6PD) via inhibition of miR-1, contributing to the switch from OXPHOS to glycolysis and the invasive property of the preneoplastic foci.

Fahrmann et al. [19] analyzed the biochemical and molecular alterations between early-stage lung adenocarcinoma and matched control tissue using integrated metabolomics and proteomics approach. They found increased glycosylation and glutaminolysis, activated nicotinic and nicotinamide salvaging pathways, and increased polyamine biosynthesis accompanied by Nrf2 activation in early-stage lung adenocarcinoma compared to control tissue. This study indicates that the interaction of Nrf2 and metabolic reprogramming has a role in malignant transformation.

In summary, a series of metabolic functions have contributed to the malignant transformation of cancer cells. Nrf2 exerts an important role in metabolism reprogramming and carcinogenesis. More investigation focusing on Nrf2-mediated metabolic reprogramming can discover the mechanisms of carcinogenesis and identify more diagnostic markers for malignant transformation.

### 4.2. Cancer Proliferation

G6PD is the first and rate-limiting enzyme in the PPP. Increased G6PD expression and activity have been observed in several cancers [44–46]. Liu et al. [47] demonstrated that G6PD was highly expressed in chronic hepatitis B virus- (HBV-) associated HCC. In the mechanism study, they found that X protein of HBV (HBx) interacts with p62 and Keap1 to form HBx-p62-Keap1 complex. In the cytoplasm, the complex hijacked Keap1 from Nrf2 leading to the nuclear translocation and activation of Nrf2. Subsequently, Nrf2 promoted the G6PD transcription and hepatocyte proliferation. These results imply a potential mechanism of HBV on the malignant transformation of hepatocytes via Nrf2-related glucose metabolism reprogramming.

B7-H3 is a member of B7 immunoregulatory glycoprotein family. The expression of B7-H3 is increased in a wide variety of cancers compared. Overexpression of B7-H3 is related to cancer progression, metastasis, and poor treatment response [48]. However, the exact underlying mechanisms are mostly unknown. Recently, Lim et al. [49] illuminated an immune-independent contribution of B7-H3 in cancer proliferation. They found that B7-H3 reduced Nrf2 transcription in breast cancer cells via an unknown mechanism. The downregulation of Nrf2 led to reduced transcription of the antioxidant targets superoxide dismutase 1 (SOD1), SOD2, and peroxiredoxin 3 (PRDX3) and increased transcription of ROS. The accumulation of ROS subsequently stabilized hypoxia-inducible factor 1 alpha (HIF1*α*), thus increasing the expression of key enzymes in the glycolytic pathway, such as lactate dehydrogenase A (LDHA) and pyruvate dehydrogenase kinase 1 (PDK1), which promoted pyruvate conversion into lactate while inhibiting pyruvate flux through the tricarboxylic acid (TCA) cycle. Metabolic imaging of human breast cancer xenografts in mice also confirmed the effects of B7-H3 in promoting glucose uptake and tumor growth. These results revealed a relationship between B7-H3/Nrf2-induced metabolic reprogramming and cancer proliferation.

As an important aspect of epigenetic regulation, microRNA is also involved in the Nrf2-induced metabolic reprogramming of cancer. Singh et al. [50] demonstrated that activation of Nrf2 signaling in cancer cells switched the carbon flux toward the PPP and the TCA cycle, reprogramming glucose metabolism via downregulation of miR-1 and miR-206. Conversely, loss of Nrf2 attenuated the level of histone deacetylase 4 (HDAC4) and increased the level of miR-1 and miR-206. Increased miR-1 and miR-206 subsequently downregulated metabolic genes and impaired NADPH production, ribose synthesis, and in vivo tumor growth. Taken together, these findings demonstrate the contribution of Nrf2 in the regulation of cancer proliferation via metabolic reprogramming and also establish the relation of miRNA regulation, glucose metabolism, and ROS homeostasis in cancer.

Nrf2 can interact with some oncogenic pathways, such as the PI3K/Akt pathway, and increase the proliferation of cancer cells via metabolic reprogramming. Mitsuishi et al. [51] showed that Nrf2 could activate genes involved in the PPP, nucleotide synthesis, and NADPH production and redirect glucose and glutamine into anabolic pathways, especially in the presence of active PI3K-Akt signaling. Forced activation of PI3K pathway increased the nuclear availability of Nrf2 and enabled Nrf2 to promote metabolic reprogramming that increases cell proliferation. The positive feedback loop between the PI3K/Akt and Keap1-Nrf2 pathway promoted the malignant evolution of lung cancer. Targeting the feedback would be an effective strategy for anticancer therapy.

Pancreatic stellate cells (PSC) is thought to be responsible for the aggressive behaviors of pancreatic ductal adenocarcinoma (PDAC) via secreting various soluble factors. Wu et al. [52] evaluated the relationship between PSC and Nrf2 and Nrf2's contribution to the progression of PDAC. They found that PSC contributed to the progression of PDAC through activation of Nrf2. As a consequence, metabolic genes involved in PPP, glutaminolysis, and glutathione biosynthesis were upregulated. Inhibition of G6PD with siRNA and chemical approaches reduced PSC-mediated cell proliferation. Among the cytokines present in PSC-conditioned media, stromal cell-derived factor-1 alpha (SDF-1*α*) and interleukin-6 (IL-6) were identified as the upstream modulators of Nrf2. In conclusion, this study reveal that SDF-1*α* and IL-6 secreted from PSC induced PDAC cell proliferation via Nrf2-activated metabolic reprogramming.

In summary, in order to sustain the rapid division of cancer, Nrf2 triggers metabolic reprogramming and provides the materials for biosynthesis.

### 4.3. Treatment Resistance

p62 is a key molecules of several critical signaling pathways [53, 54]. Increasing evidences illustrate the interaction between the p62 and Nrf2 pathway [29, 30, 55–57]. Saito et al. [58] found that metabolic reprogramming through the p62-Keap1-Nrf2 axis contributed to tumor growth and the development of drug resistance of HCC. Phosphorylated p62 can bind with Keap1 and inhibit Keap1-driven ubiquitination of Nrf2, leading to the accumulation of Nrf2 in nucleus. Subsequently, genes encoding enzymes involved in PPP, glutathione synthesis, and glutaminolysis are upregulated and cause rearrangement of glucose and glutamine metabolism. These changes stimulate proliferation potency of HCC cells and increase their tolerance to anticancer drugs. Furthermore, a small compound, K67, which inhibits Nrf2 and disrupts the interaction between phosphorylated p62-peptide and Keap1, was identified. The identified compound can sensitize cancer cells to anticancer drugs, especially in hepatitis C virus- (HCV-) positive HCC patients.

Riz et al. [59] established a carfilzomib-resistant multiple myeloma cell line (LP-1/Cfz) characterized by decreased levels of ROS, elevated levels of fatty acid oxidation, and prosurvival autophagy, while mechanistic studies demonstrated that Nrf2 is involved in metabolic reprogramming and the development of drug resistance of LP-1/Cfz. Overexpression of protein kinase R- (PKR-) like endoplasmic reticulum kinase- (PERK-) eukaryotic initiation factor 2 alpha (eIF2*α*) was detected in the resistance cell line, which caused the activation of p62. p62 competes with Nrf2 for the binding of Keap1, leading to Nrf2 activation and its target gene eukaryotic translation initiation factor 4E family member 3 (EIF4E3). The upregulation of positive feedback loop among Nrf2 and EIF4E3 led to the carfilzomib resistance of multiple myeloma cells. Genetic and pharmacologic inhibition of the Nrf2-EIF4E3 axis or the PERK-eIF2*α* pathway can sensitize the LP-1/Cfz cell to carfilzomib through modulating redox homeostasis via inhibiting fatty acid oxidation or autophagy.

Multiple studies reveal that the folate cycle is crucial to cancer-specific nutrient demands. Methylenetetrahydrofolate dehydrogenase 1-like (MTHFD1L) is a critical component of folate cycle [60–62]. As a transcription target of Nrf2, elevated MTHFD1L increased the proliferation of cancer cells in HCC via metabolic reprogramming. Knockdown of MTHFD1L caused impairment of glycolysis and metabolic changes in the TCA cycle, which impeded HCC proliferation and increased the sensitivity of HCC cells to sorafenib treatment in vitro and in vivo [63].

These studies, which focused on the Nrf2-modulated treatment resistance via metabolic reprogramming, increase our understanding on the development treatment resistance of cancer. More precise inhibitors that specifically target the Nrf2-regulated resistance will be developed in the near future.

## 5. Conclusion

**Figure 1:**
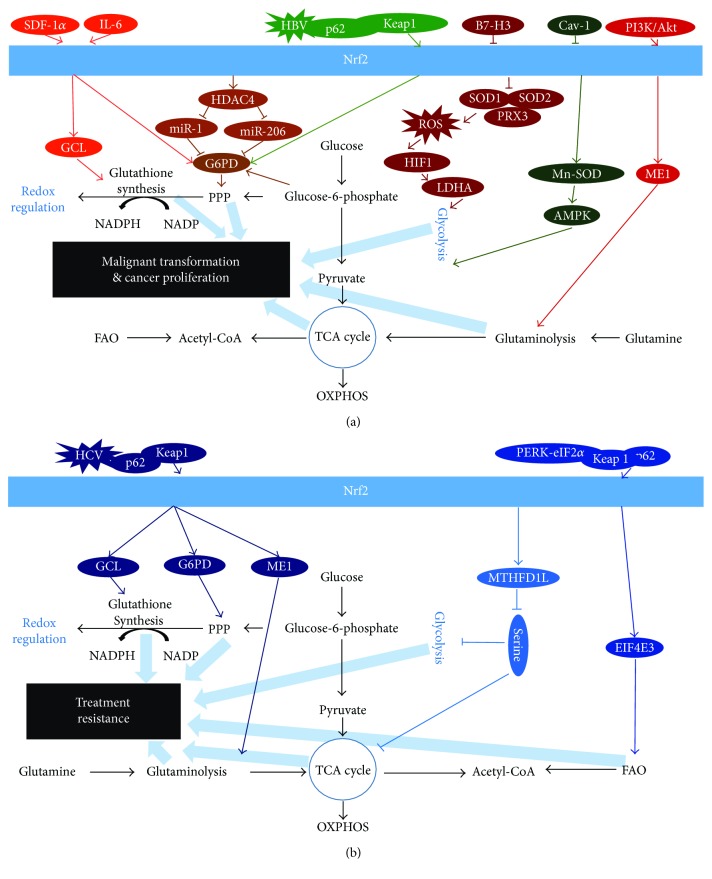
The summary of Nrf2-mediated metabolic reprogramming in cancer.

The phenomenon of cancer metabolic reprogramming is initially described by Warburg et al. in the 1920s [8]. With the development of new biochemical and molecular biological tools, the field of cancer metabolism has been intensively investigated. The spectrum of metabolic reprogramming in cancer has expanded dramatically since Warburg, especially in the recent years [2]. ROS has a huge impact on various stages of tumorigenesis. Nrf2, a key regulator of ROS, has been shown to contribute to the interplay between redox homeostasis and metabolic alternation within cancer cells [15]. In the past few years of research development, it is evident that Nrf2 not only plays a role in the malignant transformation but also contributes to cancer proliferation and the development of treatment resistance via metabolic reprogramming (Figure 1). These studies suggest that Nrf2 signaling represents a critical process in the regulation of central metabolism in cancer. Although there is still much work needed to determine the precise molecular mechanisms of Nrf2-mediated cancer metabolic reprogramming, researches focusing of Nrf2 hold the potential to discover noninvasive biomarkers and develop targeted metabolic cancer therapies.
